# Identification of virulence-related amino acid mutations of avian encephalomyelitis virus associated with vaccination methods

**DOI:** 10.3389/fvets.2025.1548515

**Published:** 2025-03-05

**Authors:** Zheng Xu, Pengcheng Fan, Chengcheng Zhang, Mengjiao Guo, Zongyi Bo, Yantao Wu, Xiaorong Zhang

**Affiliations:** ^1^Jiangsu Co-Innovation Center for the Prevention and Control of Important Animal Infectious Disease and Zoonoses, College of Veterinary Medicine, Yangzhou University, Yangzhou, China; ^2^Joint International Research Laboratory of Agriculture and Agri-Product Safety, The Ministry of Education of China, Yangzhou University, Yangzhou, China

**Keywords:** avian encephalomyelitis virus, embryo-adapted strain, mutations, vaccination method, whole-genome sequencing, phylogenetic analysis

## Abstract

Avian encephalomyelitis virus (AEV), a picornavirus, primarily infects the central nervous system of 1 to 2-week-old young chickens but not pullets. When wild-type AEV undergoes serial passaging in chicken embryos, it becomes to be embryo-adapted and can cause avian encephalomyelitis in chickens of all ages following intracutaneous infection through parenteral routes. This study was conducted to explore whether an outbreak of AEV in 95-day-old chickens was linked to inadvertent embryo adaptation of the AEV vaccine and its association with vaccination method. In this study, an AEV strain AEV/JS202201 was isolated from the flocks of chickens that had been shortly after vaccinated with the AEV vaccine combined with the avian pox vaccine by the wing-web method. Whole-genome sequencing was performed on the isolated AEV/JS202201 and the immunized VACCINE X strain. The results showed that the length of AEV/JS202201 and VACCINE X strain was determined to be 7,032 bp and 7,034 bp, respectively (both excluding the poly A tail). Compared with VACCINE X strain, one mutation, T24A, were found at the VP4 in the isolated AEV/JS202201 strain. Multiple sequence alignment revealed that no other AEV strains exhibited this mutation. Animal regression experiment confirmed that AEV/JS202201 could infect layer pullets and caused typical pathological changes in brain tissue, with a higher morbidity rate (4/10) and more severe clinical symptoms in chickens immunized via the wing-web method compared to those immunized orally (2/10). In summary, this study found a potential virulence-related mutation in the VP4 protein of AEV and emphasized that the oral vaccine method is safer than the wing-web method.

## 1 Introduction

Avian encephalomyelitis (AE), a viral disease caused by the avian encephalomyelitis virus (AEV), can affect the central nervous system of 1-2-week-old chickens, turkeys, pheasants, and quails that lack antibodies (1). Clinically, AE is characterized by ataxia and rapid tremors, and the virus is predominantly transmitted vertically through hatching eggs (2, 3). AEV is now widespread globally, causing significant economic losses due to reduced hatchability, decreased egg production, and increased early chick mortality (4, 5).

AEV is a small, non-enveloped virus, approximately 25–30 nm in diameter, classified in the family of *Picornaviridae* and the genus *Tremovirus* (6). The RNA genome of AEV was characterized by Marvil et al., comprising 7,032 nucleotides with a long open reading frame (ORF) of 6,405 nucleotides starting from the 495th position. This ORF encodes both structural and non-structural proteins, organized into three main precursor molecules: P1, P2, and P3. These precursors encode 11 proteins, including four structural proteins (VP4, VP2, VP3, and VP1) from the P1 region, and seven non-structural proteins (2A, 2B, 2C, and 3A, 3B, 3C, 3D) from the P2 and P3 regions (7, 8). Notably, the VP1 and VP2 proteins from the P1 region exhibit immunogenic properties and can serve as molecular markers for detection (9, 10).

AEV isolates are serologically similar but can be classified into two pathogenic types. The enterotropic type, represented by natural wild-type strains, infects via the oral route and can be transmitted vertically via hatching eggs, as well as causing horizontal infections in susceptible chicks (31). If the virus undergoes excessive passage in chicken embryos for vaccine production, it can infect chickens of all age, which was known as chicken embryo-adapted strain (VR strain, Van Rokel strain). The chicken embryo-adapted strain is characterized by high neurotropism and severe neurological symptoms. Generally, oral administration of VR strains does not cause infections, and they are not horizontally transmissible (11–14). Both pathological types of AEV can replicate in embryos from susceptible flocks. However, the wild-type strain rarely causes significant gross changes or severe disease, while the chicken embryo-adapted strain causes notable clinical symptoms, including encephalomalacia, muscle atrophy, and skeletal muscle solidification (15–17).

Vaccination remains the most widely employed strategy for controlling AEV and is effective in minimizing losses (18, 19). Typically, laying hens are vaccinated to create protective immunity, preventing viral transmission via eggs, while maternal antibodies also protect chicks from AEV infection during the initial 2–3 weeks (20). Both inactivated and attenuated live vaccines have been developed and utilized (21). Traditionally, avian encephalomyelitis live vaccines are administered orally through drinking water (22), effectively colonizing the intestine and providing a blockade against wild-type infections. Recently, a combined live vaccine combining avian encephalomyelitis and fowlpox has been developed and promoted for clinical use, as it addresses immunization needs for both diseases. Farms typically vaccinate flocks aged 10–16 weeks with this combined vaccine. However, to accommodate the fowlpox immunization route, wing-web inoculation is required. This vaccination method might carry a risk of inducing clinical disease, as excessive passages of the virus in chicken embryos for vaccine production can lead to adaptation, resulting in infections regardless of the chickens’ age. Glisson and Smyth have previously reported cases of AE outbreaks following the use of combined vaccines or oral vaccine strains (23, 24).

In recent years, the clinical incidence of AE has increased, with young chickens predominantly exhibiting neurological symptoms and paralysis, while older chickens show transient declines in egg production (25). The detection of novel avian encephalomyelitis strains has been rising annually, with a trend towards the emergence of dominant strains in white-feather broilers, broiler breeders, and layers. Genetic analyses reveal significant divergence between novel and classical strains (26). Currently, commercially available attenuated live vaccines provide suboptimal protection against these novel strains. Potential causes for these infections may include excessive passage of vaccines, incorrect immunization strategies, or compromised immune responses within the flocks.

In this study, we report a case of leg paralysis that occurred shortly after vaccination at 83 days of age with the combined Avian Encephalomyelitis and Avian Pox vaccine. Clinical samples from the affected chickens were collected to detect the causative pathogens, and a strain of AEV, designated AEV/JS202201, was successfully isolated. The phylogenetic characteristics and the virulence of this strain were further analyzed.

## 2 Materials and methods

### 2.1 Samples

In 2022, a farm raising Hy-Line Brown chickens reported sporadic cases of leg paralysis after vaccination at 83 days of age via the wing-web with the combined Avian Encephalomyelitis and Avian Pox vaccine. The affected chickens appeared randomly distributed within the flock. Most affected chickens displayed a crouched posture and reluctance to stand, with some lying on their sides and struggling, limping occasionally. Mixed tissue samples including brain, tendon, and visceral tissues were collected, homogenized with PBS in a mortar, and then frozen and thawed three times. The mixture was centrifuged at 3,600 rpm for 10 min at 4°C, and the supernatant was collected for RNA extraction.

### 2.2 RT-PCR

Total RNA was extracted from the clinical samples using TRIzol reagent (CWBIO, Taizhou, China) and reverse-transcribed into complementary DNA (cDNA) using EasyScript reverse transcriptase (TransGen Biotech, Beijing, China). The primers were designed with Primer 5.0 and used for AEV, MS, and ARV detection. PCR products were analyzed by 1% agarose gel electrophoresis. The primers used was listed in Table 1.

**Table 1:** Primers used in this study.

Name	Sequence
MS-F	5′-GCCATTGCTCCTTCTGTTATAGCAA-3′
MS-R	5′-TATGCTGGAAATACTGATGAAGCTTTT-3′
AEV-F	5′-TCTTATGCTGGCCCTGATCG-3′
AEV-R	5′-CTTAGCCCTTTGGTCGCACAG-3′
ARV-F	5′-ATGAGTTCGCGCAAAGTGGCTAGACG-3′
ARV-R	5′-CCCACATGTCAGCCCATTCAGAAG-3′

### 2.3 Virus isolation and identification

Six-day-old SPF chicken embryos (Boehringer Ingelheim vital bio, Beijing, China) were used for viral isolation and propagation. Brain tissue samples that tested positive for AEV by RT-PCR were homogenized with PBS and filtered through a 0.45 μm pore-size filter. A total of 200 μL of filtered supernatant was inoculated into the yolk sac of SPF embryos, while the control group received 200 μL of PBS. The embryos were incubated at 37°C and monitored daily using candling. Embryos that died within the first 24 h were excluded as non-specific mortality. After 12 days of incubation, the brain tissues of the embryonated eggs were collected and tested for AEV using RT-PCR.

### 2.4 Next-generation sequencing

Whole-genome sequencing was performed on both the VACCINE X strain and the isolated strain. Nucleic acids were extracted from brain tissue samples, fragmented, and used to construct sequencing libraries. Sequencing was conducted on the Illumina NovaSeq 6,000 platform using paired-end (PE) sequencing (Tanpu Biotech Co, Shanghai, China). Additionally, some primers were designed to amplify the specific fragments to confirm their identity.

### 2.5 Sequence alignment

Amino acid sequences of the AEV proteins were aligned using the MegAlign module in DNASTAR Lasergene 7 (DNASTAR Inc., Madison, WI, USA), and the results were visualized through ESPript 3.0.^1^ Phylogenetic trees for VP2 and the full-length viral genome were generated using the maximum likelihood (ML) method in MEGA-X, applying the general time-reversible model with gamma distribution and 1,000 bootstrap replicates. The evolutionary tree for both the full-length genome and the VP2 region were constructed using the TN93 + G + I model, with definitions on the tree. The accession numbers, countries, and definitions of sequences used in phylogenetic trees were shown in Supplementary file. The three-dimensional structure of the protein was predicted by Alphafold3 and was visualized using the PyMOL system.

### 2.6 Animal regression experiment

A total of 60 SPF chickens, aged 60 days, were randomly divided into three groups: a control group (*n* = 20), a VACCINE X group (*n* = 20), and an AEV/JS202201 group (*n* = 20). Each group was further divided according to the inoculation method, which included oral inoculation or subcutaneous injection under the wing-web. For oral inoculation, chickens were fasted for 2 h prior to receiving 100 μL of virus, administered directly into the mouth using a pipette, with their beaks gently closed to ensure swallowing. The control group was injected with 100 μL of PBS. For subcutaneous inoculation, 100 μL of virus was injected into the triangular avascular area of the wing-web using a 1 mL syringe, taking care to avoid liquid leakage. The control group was injected with 100 μL of PBS. The chickens were observed daily for clinical symptoms and morbidity rates and the results were recorded.

### 2.7 Viral shedding detection

Cloacal swabs were collected on day 3, 7, and 14. The swabs were vortexed in PBS, underwent three freeze–thaw cycles followed by centrifugation. The supernatant was collected for AEV detection using RT-qPCR. A Ct value greater than 34, corresponding to a viral copy number below 10^0.77^, was considered negative. Briefly, total RNA was extracted from cloacal swab samples using TRIzol reagent (CWBIO, Taizhou, China) and reverse-transcribed into cDNA using EasyScript reverse transcriptase (TransGen Biotech, Beijing, China). The RT-qPCR protocol followed the method of Liu et al. (27). The forward primer was 5′-GAATTAGCTCCTGGTAAACCTCG-3′ and the reverse primer was 5′-TATTATCGCAACACCCTAAGG-3′. Primers were synthesized by GenScript (Nanjing, China).

### 2.8 Histopathological examination

Two weeks later, the chickens were euthanized, and their brain tissues were collected and fixed in 10% neutral buffered formalin for 24 h to preserve cellular morphology. The tissues were trimmed, placed in embedding cassettes, and washed. Fixed brain tissues were dehydrated in a graded ethanol series to remove water, cleared with xylene, and embedded in paraffin. Sections of 4–5 μm thickness were cut using a microtome and stained with hematoxylin and eosin (H&E) to reveal cellular and tissue structure. After staining, the sections were air-dried, dehydrated, cleared, and mounted with neutral resin. The slides were examined under a light microscope to observe pathological changes, and all findings were recorded.

### 2.9 Statistical analysis

Verification was performed more than three times for all experiments. Data are presented as mean ± standard deviation (SD). GraphPad Prism software was used to determine statistical significance between groups. *p < 0.05, **p < 0.01, ***p < 0.001.

## 3 Results

### 3.1 Collection of clinical samples and identification of AEV

**Figure 1:**
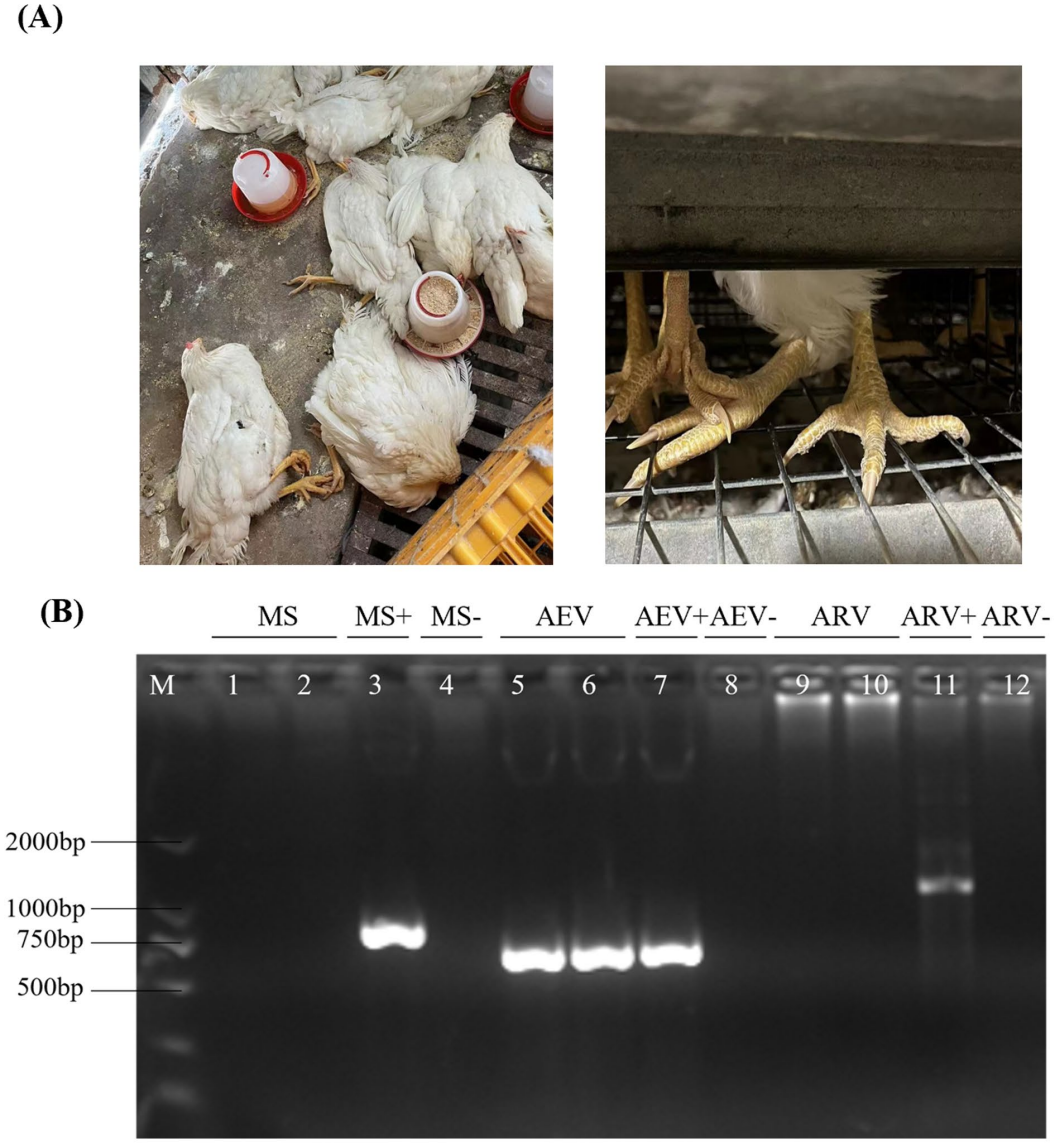
Clinical samples and the detection of AEV. **(A)** After vaccination at 83 days of age with a combined Avian Encephalomyelitis and Avian Pox vaccine, affected chickens exhibited a crouched posture and reluctance to stand, with some lying on their sides, struggling, and showing flattened proximal joints. **(B)** Mixed tissue samples, including brain, tendon, and visceral tissues, were collected for pathogen detection. MS, AEV, and ARV were tested by RT-PCR, and the results indicated the presence of AEV only, with negative results for MS and ARV.

In 2022, a farm reported cases of leg paralysis after vaccination at 83 days of age with the combined Avian Encephalomyelitis and Avian Pox vaccine. Most affected chickens displayed a crouched posture and reluctance to stand, with some lying on their sides, struggling, and flattened proximal joints (Figure 1A). To identify the pathogens involved in this outbreak, a comprehensive screening was performed for common pathogens linked to paralysis in poultry, including *Mycoplasma Synoviae* (MS), Avian Reovirus (ARV), and Avian Encephalomyelitis Virus (AEV). Mixed tissue samples, including brain, tendon, and visceral tissues, were collected for pathogen detection. The results showed that AEV was the only pathogen detected (Figure 1B). These findings suggest that the paralysis observed in chickens was likely due to AEV infection.

### 3.2 Virus isolation

**Figure 2:**
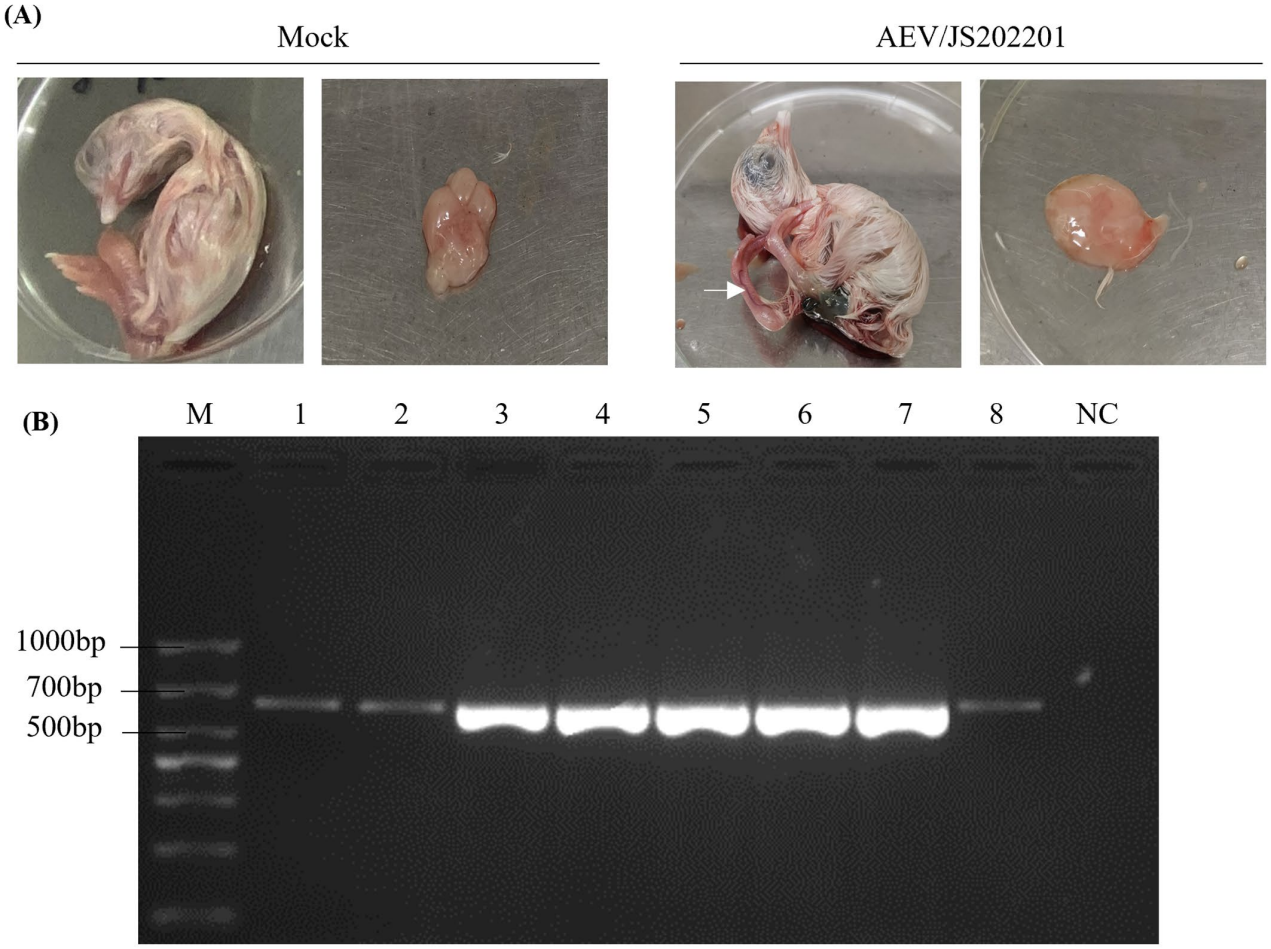
Isolation of AEV/JS202201. **(A)** Six-day-old SPF chicken embryos were inoculated with supernatants from AEV-positive brain tissue samples via the yolk sac route, while PBS-injected embryos served as the control group. After 12 days of incubation, AEV-infected embryos exhibited typical pathological changes, including leg rigidity and encephalomalacia, as indicated by the white arrow. **(B)** Brain tissue samples from embryos infected with AEV/JS202201 (line 1–8) were detected using RT-PCR.

To isolate the AEV, supernatants from AEV-positive brain tissue samples were inoculated into 6-day-old SPF chicken embryos via the yolk sac pathway. After 12 days of incubation, embryos were collected on day 18, and no mortality was observed. As shown in Figure 2A, AEV-infected embryos exhibited typical pathological changes, including leg rigidity and encephalomalacia. Further testing of brain tissues from these embryos using RT-PCR confirmed the presence of AEV (Figure 2B). These findings confirm the successful isolation of the AEV. This strain, which displayed symptoms characteristic of the VR strain in SPF embryos, was designated as AEV/JS202201.

### 3.3 Phylogenetic analysis of the sequences of AEV

**Figure 3:**
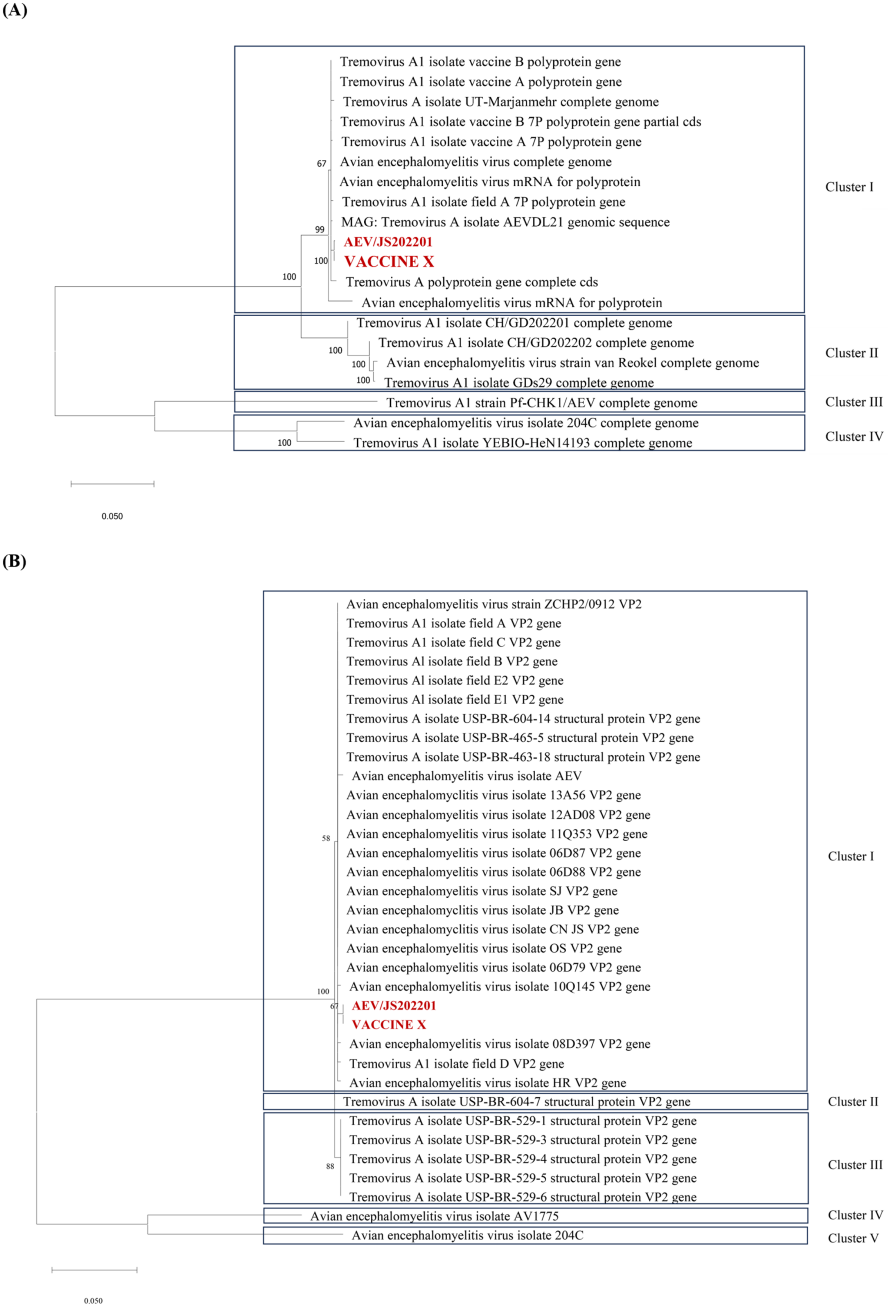
Phylogenetic tree analysis of the AEV/JS202201 and VACCINE X strain. **(A)** Phylogenetic tree of AEV strains based on full-length genome sequences. The maximum likelihood (ML) tree was constructed using 20 complete genome sequences available in GenBank. **(B)** Phylogenetic analysis of the VP2 gene sequences. The ML tree was constructed using 34 VP2 gene sequences.

To explore the genetic characteristics of AEV/JS202201, the whole-genome sequences of AEV/JS202201 and the immunized VACCINE X strain were performed. The genome sequence was deposited in GenBank under Accession Number PQ463256. The results showed that the genome lengths of the VACCINE X and AEV/JS202201 were 7,034 bp and 7,032 bp, respectively (excluding the polyA tail). Both genomes contained a single large ORF of 6,405 nucleotides, encoding 2,135 amino acids, with no insertions or deletions. To analyze the evolutionary relationships among AEV strains, the maximum likelihood (ML) phylogenetic trees based on the full-length genome sequences were constructed. It was found that AEV strains can be divided into four clades based on the full genome sequences (Figure 3A). The first clade included strains from the USA, UK, China, and Iran, while the second and fourth clades only consisted of Chinese strains, and the third clade contained only the Hungarian strain. Both VACCINE X and AEV/JS202201, marked in red, were grouped within the first clade. Similarly, the ML tree based on VP2 sequences placed VACCINE X and AEV/JS202201 in the first clade (Figure 3B).

### 3.4 Sequence analysis

**Figure 4:**
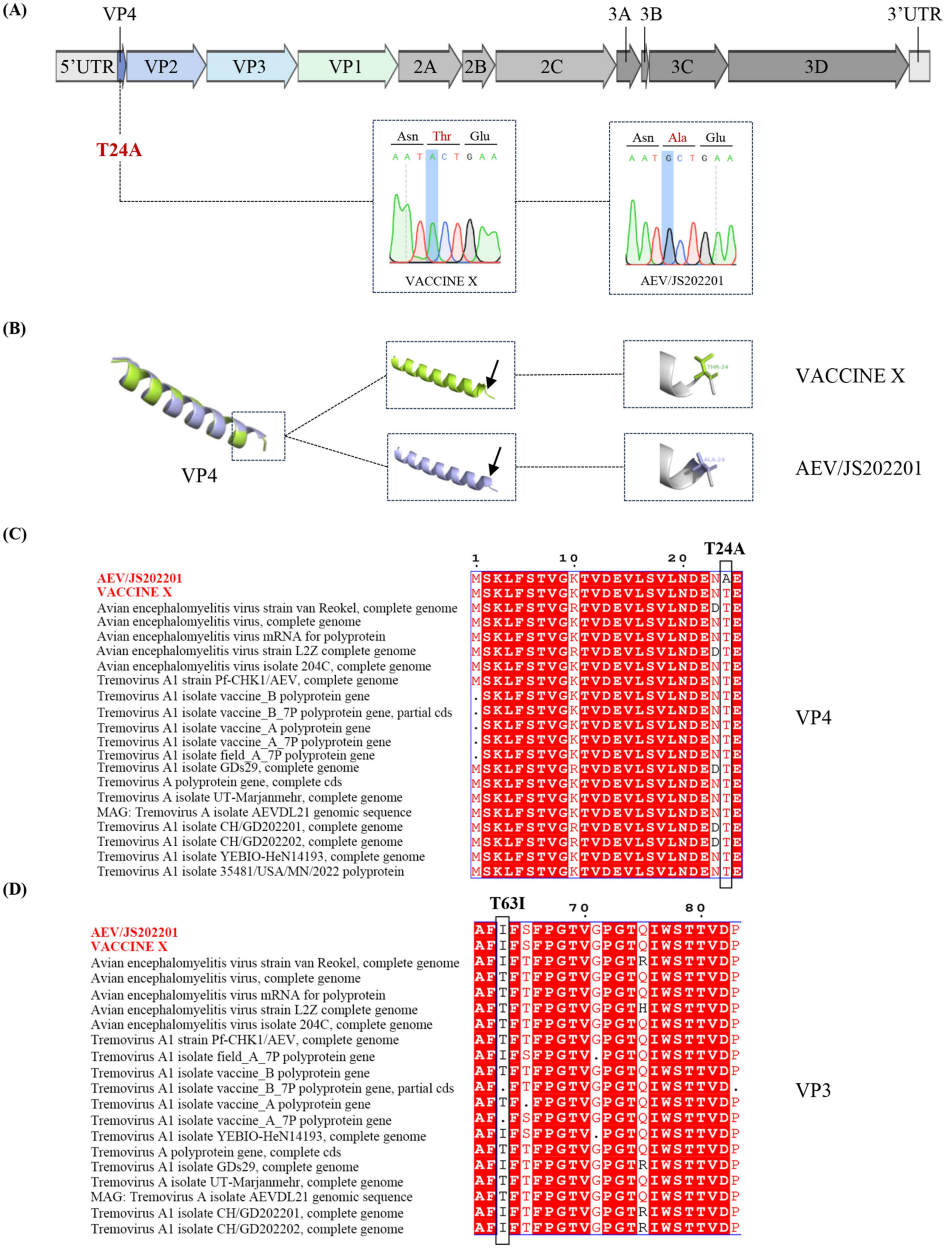
Genetic sequence analysis of the AEV/JS202201 and VACCINE X strain. **(A)** Genome structure and mutations in AEV/JS202201 compared to VACCINE X strain. **(B)** 3D Structural comparison of VP4 protein. Three-dimensional structural of the VP4 protein from VACCINE X and AEV/JS202201 were modeled by Alphafold3. The monomer structural overlap of the VP4 protein between the two strains was visualized using PyMOL. **(C)** Twenty representative strains of AEV were chosen for the analysis. The mutation from threonine (T) in VACCINE X to alanine (A) in AEV/JS202201 at position 24 in the VP4 protein was shown. **(D)** Twenty representative strains of AEV were chosen for the analysis. Threonine (T) in both VACCINE X and AEV/JS202201 mutated to isoleucine (I) at amino acid position 63.

To investigate the differences between VACCINE X and AEV/JS202201, their structural proteins (VP1, VP2, VP3, and VP4) and non-structural proteins (2A-2C, 3A-3D) were compared. A single amino acid mutation from threonine (T) in the VACCINE X to alanine (A) in AEV/JS202201 at amino acid position 24 in the VP4 region was found (Figures 4A,C), due to a nucleotide mutation from A in the VACCINE X mutated to G in AEV/JS202201 at the position 70. To further characterize the structural implications of this amino acid mutation, the VP4 protein of VACCINE X and AEV/JS202201 was modeled by Alphafold3 and visualized using the PyMOL system. The T24A mutation resulted in a prominent structural change, altering the secondary structure from a coiled conformation to a helical structure (Figure 4B).

Based on previously reported mutations associated with embryo adaptation (VP2-Q184R, VP3-T63I) (28), we compared these sites between VACCINE X and AEV/JS202201 and found that no relevant SNPs were detected in VP2. However, we observed the mutation from threonine to isoleucine at position 63 in the VP3 region (Figure 4D).

### 3.5 Animal regression experiment

**Figure 5:**
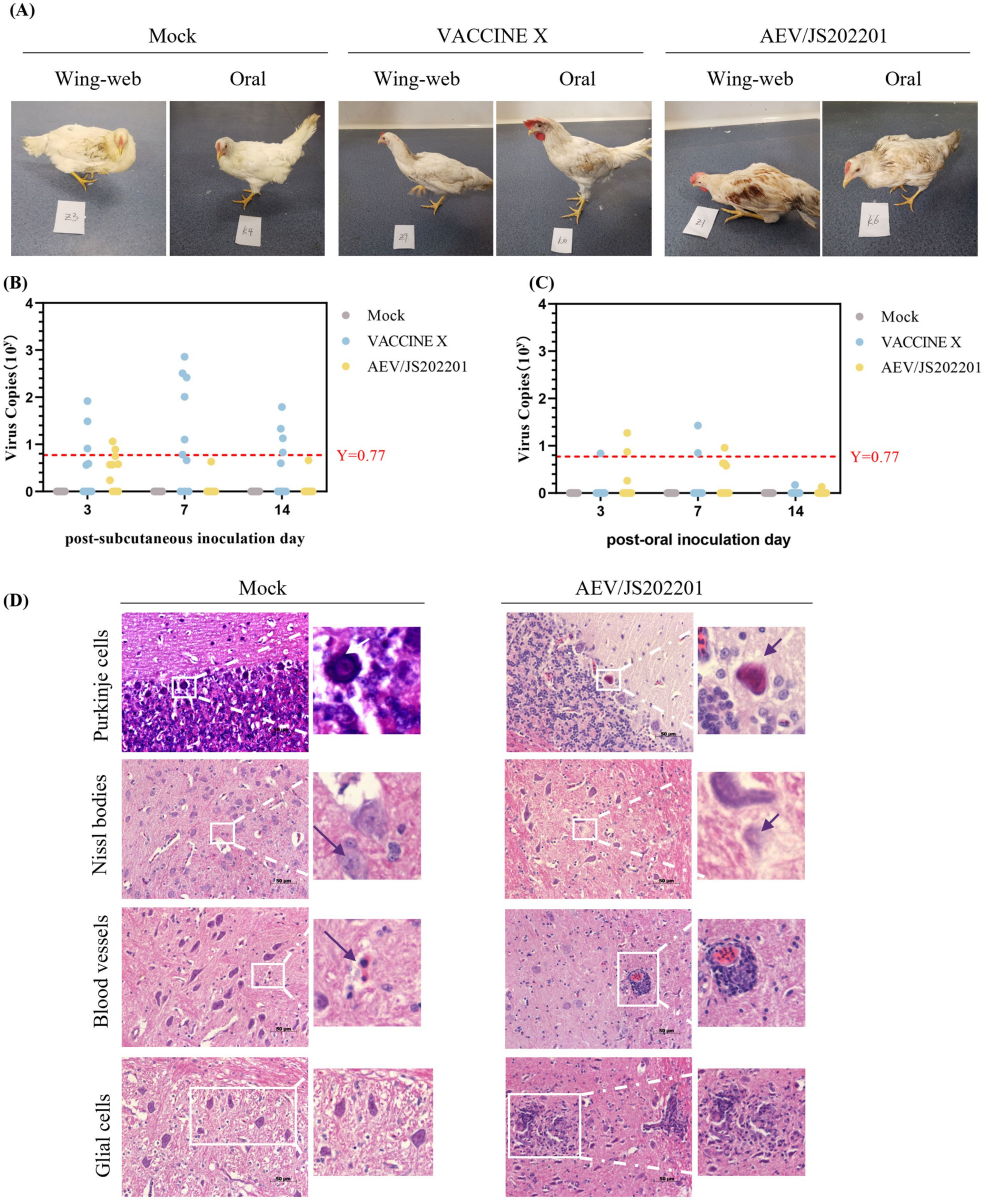
Animal regression experiment. **(A)** Representative clinical signs in chickens inoculated with AEV/JS202201 and VACCINE X, including depression, ataxia, and tarsal sitting. **(B)** Viral RNA load in cloacal swabs from chickens in subcutaneous inoculation groups (PBS, VACCINE X, and AEV/JS202201) was tested on days 3, 7, and 14 post-inoculation. The red horizontal line represents the negative threshold, corresponding to a viral copy number of 10^0.77^. **(C)** Viral shedding analysis in oral inoculation group (PBS, VACCINE X, and AEV/JS202201). The red horizontal line represents the negative threshold, corresponding to a viral copy number of 10^0.77^. **(D)** Brain tissues from chickens inoculated with AEV/JS202201 displayed significant histopathological change.

To evaluate the virulence of AEV/JS202201, an animal challenge experiment was conducted using 60 chickens, divided into three groups and inoculated either orally or subcutaneously under the wing-web with PBS, VACCINE X, and AEV/JS202201. Details of groupings, inoculation methods, doses, and the morbidity are provided in Table 2. In the oral inoculation group, no clinical signs were observed in either the control or VACCINE X groups, while 2 chickens in the AEV/JS202201 group exhibited mental depression. In the subcutaneous inoculation group, no symptoms were detected in the PBS group. However, one chicken in the vaccine group displayed signs of illness, and 4 chickens in the AEV/JS202201 group showed pronounced symptoms, including depression, ataxia, and tarsal sitting. The represent clinical signs were shown in Figure 5A. Collectively, the AEV/JS202201 group exhibited more severe clinical symptoms and morbidity than the VACCINE X group, and a higher morbidity was shown in wing-web groups in both VACCINE X and AEV/JS202201 groups.

**Table 2:** Morbidity rate table for animal regression experiments.

Group	Num.	Inoculation method	Affected	Morbidity
Control	10	Oral	/	/
	10	Subcutaneous	/	/
Vaccine	10	Oral	/	/
	10	Subcutaneous	1	10%
AEV/JS202201	10	Oral	2	20%
	10	Subcutaneous	4	40%

To evaluate viral shedding following challenge, viral RNA in the cloacal swabs, collected from the chickens on days 3, 7, and 14 post-inoculation, was tested using RT-qPCR. As shown in Figure 5B, the VACCINE X group exhibited a higher viral load and positive rate than AEV/JS202201 group in subcutaneous inoculation group. Additionally, chickens in VACCINE X group showed the highest viral load at 7 dpi, while the AEV/JS202201 group had the highest viral load at 3 dpi and could hardly be detected at 7 and 14 dpi. As shown in Figure 5C, in terms of oral inoculation, the viral load and positive detection in VACCINE X group were lower than in subcutaneous group. In AEV/JS202201 oral-inoculation group, the viral load and detection rate was at a similar level compared to subcutaneous group.

Finally, to assess whether AEV/JS202201 could induce brain lesions in chickens, brain tissues from the experimental groups were collected and examined histopathologically. Compared to the control group, AEV/JS202201-infected chickens exhibited characteristic lesions, with hypertrophy, deformation, dissolution, and necrosis in Purkinje cells. Under normal circumstances, Nissl bodies are typically granular and distributed within the cytoplasm. After infection with AEV/JS202201, Nissl bodies were observed to dissolve. During infection, there was a significant increase in the number of lymphocytes, which accumulated to form “cuffs” around blood vessels. Additionally, microglial proliferation was observed, forming both diffuse and nodular aggregates, contrasting with the relatively stable number and scattered distribution of microglial cells in the control group (Figure 5D). These findings demonstrate that the AEV/JS202201 strain induces significant pathological changes in chicken brain tissue.

## 4 Discussion

Since its first recorded outbreak in the United States in 1932, AE has become a globally prevalent disease, spreading across Africa, Asia, Australia, Europe, and the Americas. A study conducted in Bangladesh reported a 70.18% positive rate of AEV antibody in 275 serum samples from 39 unvaccinated chickens (6). In China, AE was first reported in Guangdong Province in 1980 (29). In 2022, a study reported a 46.26% positive rate in 294 clinical samples across Guangdong and Jiangxi province in China (30).

In this study, a farm raising Hy-Line Brown chickens reported sporadic cases of leg paralysis after wing-web vaccination at 83 days of age with the combined Avian Encephalomyelitis and Avian Pox vaccine. Most affected chickens exhibited a crouched posture and reluctance to stand, with some lying on their sides, struggling, and occasionally limping. These symptoms significantly impacted the movement, feeding and broiler growth performance of chickens. Pathogens that cause leg disorders in chickens were detected, including avian encephalomyelitis virus (AEV), avian reovirus (ARV), and *Mycoplasma synoviae* (MS). The results indicated that AEV was the causative pathogen. Subsequent viral isolation and identification efforts led to the successful isolation of AEV/JS202201 strain. This AEV/JS202201 strain induced characteristic pathological changes in 6-day-old SPF chicken embryos following yolk sac inoculation, such as leg stiffness and encephalomalacia (Figure 2A). The whole-genome sequences of AEV/JS202201 and VACCINE X strain were determined to further analysis their genetic characteristics. Phylogenetic analysis, based on both the full genome sequences and the VP2 sequences, placed them into the first clade (Figure 3).

After comparing the amino acid sequences of AEV/JS202201 and VACCINE X, a mutation in the VP4 (T24A) of AEV/JS202201 was identified, which resulted in a change of the protein’s secondary structure from a random coil in VACCINE X to an *α*-helix in AEV/JS202201. Additionally, the sequence alignment of VP4 showed that all other strains did not contain this mutation (Figure 4C). Previously, two single nucleotide polymorphisms (SNPs) associated with embryo adaptation were identified, which lead to amino acid mutations G184A in VP2 and T63I in VP3 (28). In our study, the G184A mutation in the VP2 region was not found in either AEV/JS202201 or VACCINE X. However, the mutation T63I was observed in VP3, consistent with previous findings (Figure 4D).

Animal regression experiments revealed a 40% (4/10) morbidity rate in chickens subcutaneously inoculated with AEV/JS202201, significantly higher than that observed with its oral inoculation group (2/10). Notably, one chicken in VACCINE X wing-web inoculation group exhibited signs of illness, while no clinical signs were observed in VACCINE X oral inoculation group. Histopathological examination showed typical lesions, including perivascular lymphocyte infiltration, microglial nodules in the cerebellar molecular layer, and degenerative changes in Purkinje cells in AEV/JS202201 group (Figure 5D).

Based on the animal experiments, the T24A mutation in VP4 protein was speculated to contribute to the increased virulence of AEV/JS202201 strain, as it was the only mutation identified in AEV encoded proteins compared to VACCINE X strain. Additionally, it was speculated that AEV/JS202201 might be an embryo-adapted strain that induces classical clinical signs and pathological changes in both SPF embryos and older chickens. Moreover, the study also suggested that the oral inoculation may be a safer way to immunize the AEV vaccine, as a higher morbidity was observed in wing-web immunization method in both AEV/JS202201 and VACCINE X group. Interestingly, VACCINE X strain also caused low morbidity (1/10) via subcutaneous inoculation, possibly due to the embryo-adapted T63I mutation in the VP3 protein. This highlights the need for greater attention in AEV vaccine preparation. Although the T24A mutation provides new insight into AEV virulence, further studies using a reverse genetics platform are necessary to confirm its role.

This study successfully isolated and sequenced a virulent strain of AEV, identifying a novel T24A mutation in the VP4 protein. Animal regression experiment confirmed that the isolated AEV/JS202201 could infect chickens beyond the typical susceptible age, resulting in a 20% morbidity rate through oral infection and a 40% morbidity rate via subcutaneous inoculation. Aberrantly, the infection of VACCINE X strain also inducing 10% morbidity via subcutaneous inoculation, which might due to the existence of the embryo-adapted T63I mutation in the VP3 protein. Collectively, this study identified a potential virulence-related mutation in VP4 protein of AEV, and demonstrated that oral immunization method is a more safer vaccination method compared to the wing-web intracutaneous approach.

## Data Availability

The data presented in the study are deposited in the GenBank repository, accession number PQ463256.

## Footnotes

^1^ https://espript.ibcp.fr/ESPript/ESPript/

## Supplementary material

SUPPLEMENTARY TABLE S1 The information of the AEV strains used in each Figure.
